# Learning From Experience and Finding the Right Balance in the Governance of Artificial Intelligence and Digital Health Technologies

**DOI:** 10.2196/43682

**Published:** 2023-04-14

**Authors:** Stephen Gilbert, Stuart Anderson, Martin Daumer, Phoebe Li, Tom Melvin, Robin Williams

**Affiliations:** 1 Else Kröner Fresenius Center for Digital Health Technische Universität Dresden Dresden Germany; 2 School of Social and Political Science University of Edinburgh Edinburgh United Kingdom; 3 School of Computation, Information and Technology Technische Universität München Munich Germany; 4 School of Law, Politics and Sociology University of Sussex Brighton United Kingdom; 5 School of Medicine Trinity College Dublin Dublin Ireland

**Keywords:** artificial intelligence, machine learning, regulation, algorithm change protocol, health care, regulatory framework, medical tool, tool, patient, intervention, safety, performance, technology, implementation

## Abstract

Artificial intelligence (AI) and machine learning medical tools have the potential to be transformative in care delivery; however, this change will only be realized if accompanied by effective governance that ensures patient safety and public trust. Recent digital health initiatives have called for tighter governance of digital health. A correct balance must be found between ensuring product safety and performance while also enabling the innovation needed to deliver better approaches for patients and affordable efficient health care for society. This requires innovative, fit-for-purpose approaches to regulation. Digital health technologies, particularly AI-based tools, pose specific challenges to the development and implementation of functional regulation. The approaches of regulatory science and “better regulation” have a critical role in developing and evaluating solutions to these problems and ensuring effective implementation. We describe the divergent approaches of the European Union and the United States in the implementation of new regulatory approaches in digital health, and we consider the United Kingdom as a third example, which is in a unique position of developing a new post-Brexit regulatory framework.

## Introduction

The speed of development of digital health technologies (DHTs) and artificial intelligence–enabled medical devices (AIeMDs) has led to a degree of concern regarding the oversight of these technologies as well as calls for stronger governance oversight [1-3]. AIeMD and DHT pose specific challenges to regulatory oversight [4]. Traditional regulatory approaches have paid little attention to unintended consequences, which may inhibit the evolution and uptake of potentially beneficial technologies. If an area offers high potential benefits to patients, health care systems, and the wider economy, then its degree of technological innovation and the new oversight challenges these developments pose must be matched by the effort and resources applied to the design and implementation of fit-for-purpose regulation. This is good for patients, for further development of technology, and public acceptance of these innovations. AIeMD and DHT are examples of technologies for which the establishment of trustworthiness and accountability to patients, health care providers, and health care systems is particularly essential, as both ethical challenges and potential benefits are substantial [3,5].

This paper has been informed by multistakeholder interactive UK Research and Innovation (UKRI)–funded workshops, which explored the optimal post-Brexit regulatory approach for the UK governance of AI-based DHTs [6]. They involved health care professionals, regulators, health care providers, health care system digitization bodies, regulatory science and law academics, regulatory specialists, and consultants, with consideration of the viewpoints of patient groups.

### Terminology

The precise definitions and terms for medical device software in the AIaMD and wider DHT area differ between countries. The focus of this paper is on standalone digital tools; AI and digital approaches are often also used in hardware medical devices. The International Medical Device Regulators Forum (IMDRF) proposes machine learning-enabled medical devices as a subset of artificial intelligence–enabled medical devices (AIMDs) [7]. We agree that this term is appropriate; however, the abbreviation AIMD is used in the European Union in the context of active implantable medical devices [8], so this can lead to confusion. Other sources use the term AI as a medical device (AIaMD) [9]; however, this term may be confusing in the context of whether the medical device’s principal functional approach is through AI or whether AI is an additional or minor part of the device’s overall function. For this reason, we use the term AI-enabled medical device and the abbreviation AIeMD.

## Challenges and Opportunities

### Why “Better Regulation” Is Needed and Not More or Less Regulation

The political movement for “Brexit” had a prominent manifesto for “cutting the red tape” [10]. The domain of AIeMD and DHT regulation may not be an obvious area for deregulation. Inappropriate deregulation could risk patient safety and undermine regulatory convergence, thereby increasing manufacturer workload and interfering with bidirectional market access and trade of these medical devices between the United Kingdom and the European Union. Considering the “tightness” of regulation in the AIeMD and DHT sectors, it is also important to consider the internationally divergent approaches, as outlined in depth below. In the last decade, the United States has adopted a more flexible approach than the United Kingdom and the European Union [11,12]. There are well-argued concerns about increasing “red tape” limiting the ability of innovation in AIeMDs and DHT to deliver functional and helpful technologies to health care providers and systems [13]. Legislators have to balance approaches that would ensure strong oversight of patient safety, in addition to those that bring important new advancements in diagnosis or treatment and meet citizens’ expectations for the provision of the international state of the art in health care. Some new AIeMD and DHT technologies may have the potential to deliver transformative gains in the efficiency of health care system [3,5]. Many citizens, particularly the young, also care about having access to the latest technological developments and convenient modern digital solutions (eg, app-based health DHTs, many of which are classified as medical devices). On the level of national policy, legislators have to balance promoting national industrial competitiveness with international requirements for free trade alongside health benefits. The current EU medical devices regulation (MDR) legislation already enshrines this duality and aims to provide [14]:

a high level of protection of health for patients and users, and taking into account the small- and medium-sized enterprises that are active in this sector...Both objectives are being pursued simultaneously and are inseparably linked whilst one not being secondary to the other.

**Figure 1:**
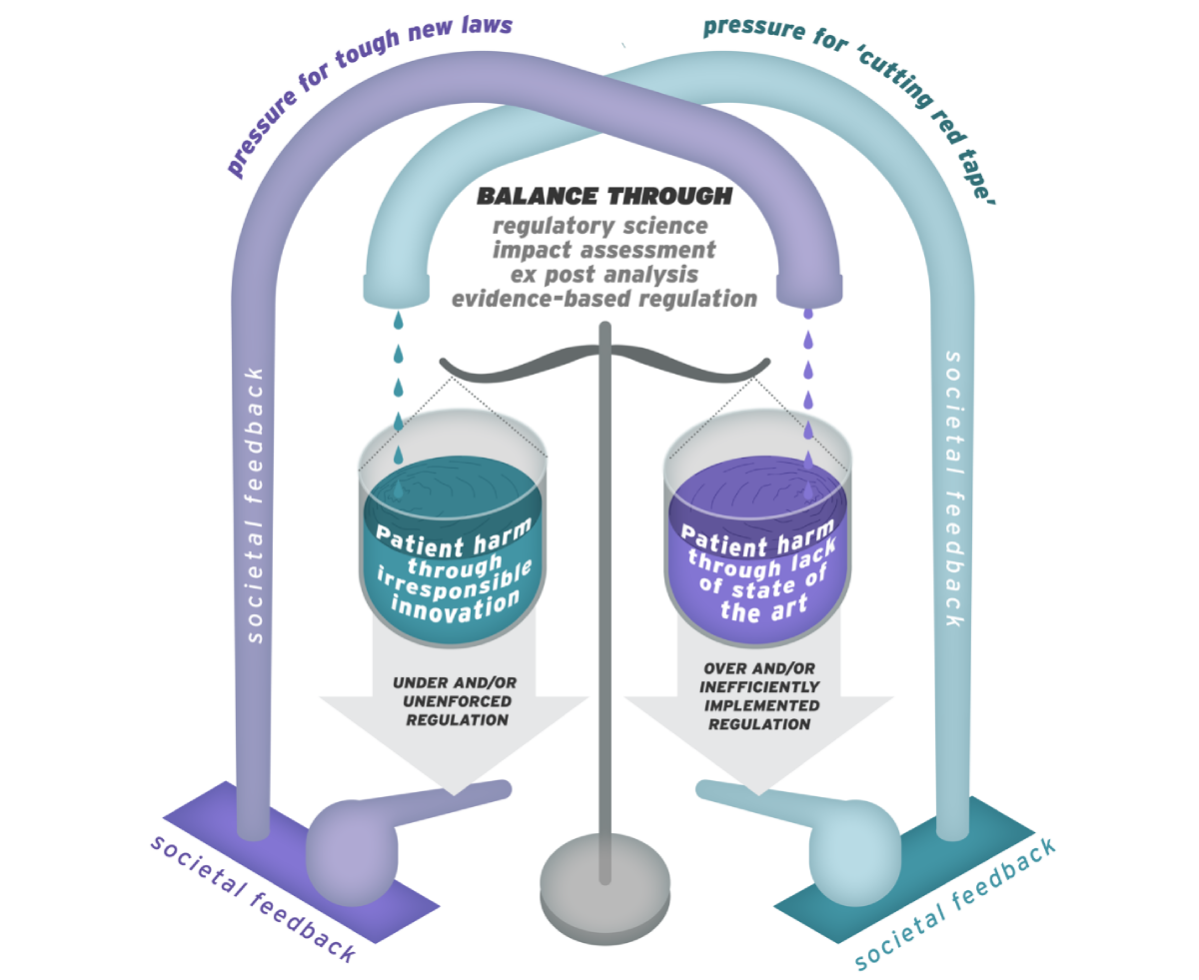
Societal level feedback loops in regulation (figure concept developed by Stephen Gilbert, figure graphic design by Andrew Berry).

In general, populations are in favor of the careful regulation of quality in critical health sectors [15,16], but when regulations limit the availability of essential or lifesaving materials or products, there is strong public pressure for increased product availability [17,18]. Governments and regulators have a duty to manage contradictory societal expectations. Overzealous, badly implemented, poorly targeted, nonadapting, or inefficiently delivered regulations can, through blocking access to essential products, have inverse consequence of their intentions [19]. Underregulation can lead to the following: insufficient oversight, irresponsible manufacturers paying insufficient attention to safety, and patient harms, resulting in public and political pressure for tighter regulation [20]. These opposing forces can, in the medium term, lead to a tendency for regulatory policy to sway between excessively restrictive regulation and insufficient oversight. We argue that through intelligent, innovative, and well-resourced regulation, based on regulatory science, this imbalance and counterproductive cycle can be avoided (Figure 1).

**Figure 2:**
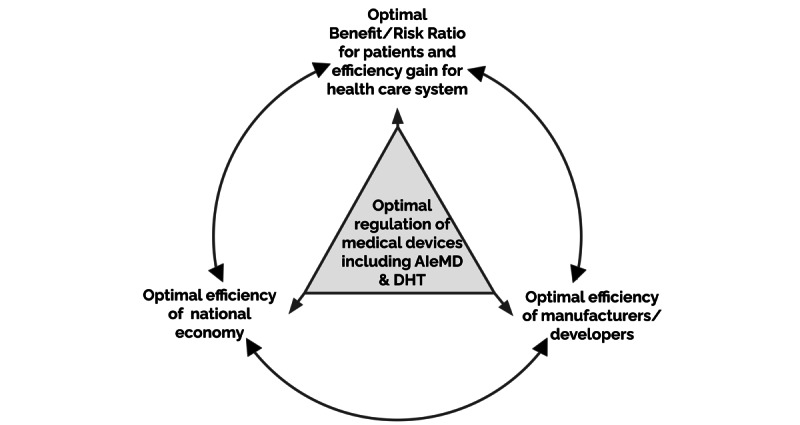
Balancing goals for optimal regulation of medical devices. AIeMDs:artificial intelligence–enabled medical devices; DHT: digital health technologies.

We argue that regulations can be developed based upon evidence-based principles and with innovation combined with oversight to ensure patient safety while simultaneously enabling new market entry and promoting new thinking within established companies. This concept is shown in Figure 2. In this way, well-balanced and well-designed regulation promotes innovation to meet patients’ needs, benefits health care, and benefits society as a whole, through the support of the economy—these stakeholders’ interests are not as antagonistic as often presented.

### EU Medical Device Regulation and its Impact on AIeMD and DHT

The 2017 EU Regulations for medical devices (MDR) governs the area of AIeMD and medical device DHT and sets out detailed requirements and obligations for product developers [14]. The MDR had a large impact on the regulation of AIeMD and DHT, and it is an important case study for the potential negative impacts of regulation on innovation [21,22]. The most acute impact of the EU MDR on the DHT sector has resulted from the systematic shortage of regulatory capacity and unreadiness of systems [23-25]. The combination of many new requirements on manufacturers and the near impossibility to meet these requirements due to unavailable critical infrastructure is resulting in a failure of market availability of products and the stifling of EU innovation [21,22,25-29]. Anecdotally, the increased requirements of MDR have already resulted in a shift in companies’ regulatory market approval strategy for products from an EU-first approach to a policy of first seeking US approval [27]. This effect is particularly acute for innovative AIeMDs and DHTs, as the measures that have been recently proposed to ease the backlog of “legacy” MDR approvals, that are likely to soon become law, do not address the approval of new devices. If enacted, these proposals will allow older devices to stay on the market if manufacturers declare that their devices are Device “in transition” to MDR, for example, they have registered their readiness for notified body (NB) audit and that they have adapted their quality management system, vigilance, and post market surveillance processes to MDR requirements [19,25,30-32]. The proposed changes could allow older devices to remain on the market, under transitional provisions (before they gain MDR approval) until up to December 31, 2028, for lower class risk devices [31]. This proposal could have an indirect effect of easing of the capacity bottleneck for innovative AIeMDs and DHTs also, as there may be reduced urgent demand for NBs from the developers of older devices. It remains to be seen to what extent this recent development will assist with the critically difficult situation that these innovative developers face, generally through no fault of their own.

The more stringent requirements of MDR on manufacturers are in part due to the mechanism described in Figure 1. The pre-MDR EU regulatory framework was viewed by some as having insufficient transparency and controls, particularly for the oversight of conformity assessment, clinical evidence, and post market surveillance [12,33]. A number of implant device recalls and evidence of illegal activity in implant manufacture gained media and public attention [20]. Clinical evidence requirements were increased in a revision of guidelines [34], and a new regulatory regime was welcomed by some [24], but early questions were also raised regarding overregulation and the risk of implementation delays and nonreadiness of critical infrastructure [27,35]. These focused particularly on the NB system of EU regulatory oversight and delivery.

We argue that the implementation delays and lack of capacity diminish trust in governance, which is critical for the societal and political support of regulation. It is therefore critical for the AIeMD and DHT sector that a similar scenario is avoided with future programs of regulation, particularly, as there are 3 proposals for regulations passing through the EU legislative process, the Artificial Intelligence Act, the European Health Data Space, and the Digital Markets Act [36-38]. NBs have flagged the serious risk that the EU AI Act will see a recurrence of these, given the increased expert capacity needed to meet its substantial additional procedures and the complexity of the interaction of different NB expertise areas [39,40].

### Better Regulation

How could this situation be avoided for future regulation of medical devices, and particularly for the in-the-pipeline new regulation of AIeMD, and what lessons could be learned by other countries introducing new AIeMD regulation, for example, in the United Kingdom? It is of note that, over recent years, the United Kingdom and European Union have been building processes for better regulation [41,42], the application of which is now required for new EU regulations. If these principles and processes had been well applied to MDR, they may have prevented or at least warned of the impending capacity risks and risks nonreadiness of the regulatory system. The use of regulation impact assessment, a main pillar of better regulation, is not guaranteed; however, the application of MDR [43] would have been challenging. The critical implementation problem has been NB capacity, and it would have been difficult to assess whether sufficient independent actors were likely to commit to the arduous approval process to become MDR-approved NBs. Nonetheless, the following principles, which are in line with better regulation principals [41,42], should guide the introduction of future regulation (particularly of AIeMD), and better answer the question of who regulates the regulator. Legislators should ensure that the following are carried out: (1) better “risk assessment” of the regulation (ie, impact assessment after the “intended purpose” of regulation has been clearly defined); (2) a “conformity assessment” of regulation to some underlying “performance requirements” by an independent oversight body or board, that is, to oversee the approval of regulation, so that it faces “scrutiny” from outside; (3) “post market surveillance”/“real-world data collection”/“real world performance monitoring” of regulation performance for near real-time assessment of the functionality and efficiency of regulatory implementation, that is, ex-post analysis; and (4) in the case of “incidents” in the implementation of regulation, there should be a “root cause analysis” of the “corrective and preventive actions,” that is, methods for evaluating causal effects, with greater accountability of the regulatory process to its effects [41].

How could this situation be avoided for future regulation of medical devices, and particularly for the in-the-pipeline new regulation of AIeMDs, and what lessons could be learned in other countries introducing new AIeMD regulation, for example, in the United Kingdom? It is highly likely that there will be a large number of new and on-market AIeMDs that will require approval, given the current rate of growth of the sector. It is of note that, over recent years, the European Union has been building a process and toolbox for better regulation, the latter of which was published in 2021, and its application is now a formal requirement for new EU regulations. The United Kingdom has a similar approach through interim guidance [42]. If these principles and processes had been well applied to MDR, they might well have prevented many aspects of its implementation failure. A particularly important aspect would have been the requirement for an impact assessment of the new regulation. This impact assessment would, admittedly, have been immensely challenging to conduct, as the critical factor in the functioning of EU MDR and EU in vitro diagnostic devices regulation (IVDR) is that sufficient independent actors decide to invest and undergo an arduous approval process to become approved NBs, so as to provide the required NB capacity in a timely manner. Our specific suggestions of how to mitigate EU MDR and IVDR implementation challenges are presented in Textbox 1; however, a detailed ex*-*post analysis following the better regulation toolbox is needed to better structure lessons learned and preventions.

**Suggestions of how to mitigate EU medical device regulation and in vitro diagnostic devices regulation implementation challenges and how these could have been avoided.**

**Problem**

- IT systems not ready
- Notified body readiness

**Implications**

- Uncertainty
- Unless workarounds provided, long delays to approval, with implications for patient care, health system efficiency, and European Union competitiveness

**Prevention**

- Advance planning in legislation that IT systems are optional until solutions complete
- Increase incentives for notified bodies (NBs)
- Phased introduction of new NB responsibilities
- Well-planned phased introduction by device type
- Well-planned phased introduction of manufacturer requirements

**Possible mitigation measures**

- Temporary workarounds supported by Medical Device Coordination Group Guidance or implementing acts where needed
- Eventually, after several years of calls for action from stakeholders, the action was taken by regulators and legislators to propose mitigation measures of this type [19,31,32]

Ultimately, there may be no perfect answer to the question of “who should regulate the regulator.” Any independent oversight body or board risks the inclusion of an additional layer of complexity between the regulators and the legislators, and it would be challenging to decide on who such a board would include to optimally deliver its role. Meaningful performance requirements to assess the delivery of a complex regulation like MDR would also be difficult to define a priori when implementation is so dependent on external political, legal, and economic factors. It may be that the 2027 European Commission (EC) review of MDR will deliver the required oversight and review. The announcements and proposals for revisions of December 2022 and January 2023 do show that, eventually, the legislators and regulators are responsive to stakeholder pressure and the reality of on-the-ground implementation challenges [19,31].

### The Future of EU AIeMD Regulation

There is an expert opinion that AIeMD needs to be better regulated in the European Union [4,44]. The EU medical device directive (MDD) was replaced by MDR, and although there have been changes in the risk classification of software, there remains no definition of AIeMD in the text of the regulation or the current guidance documents [4]. This may be the result of an important linked cross-sectoral regulation currently in the EU legislative process, the EU AI Act [4,36]. The Netherlands government commissioned a detailed legal report which concluded that, although AIeMD needs to be better regulated, the proposal AI Act is not the best solution and is not legally compatible with many aspects of the MDR and IVDR [44]. Our view is that the gaps are not best addressed through new legislation that risks unintended consequences similar to those described here for MDR. Alternatives to new legislation for the EU regulation of DHT and AIeMD exist. There are three main mechanisms through which the European Union could remedy shortcomings and gaps in the current regulation of AIeMD: (1) through an update to the MDR, perhaps in its 2027 timetabled legislative review; (2) through common specifications, as implementing legislation under MDR, for AIeMD; and (3) through specific detailed guidance for AIeMD. The EU-MDR includes a mechanism for binding common specifications applicable to categories of devices, which do not have detailed pre-existing harmonized standards. A common specification could be published for AIeMD, setting out specific approaches and requirements. A second nonlegislative approach, which we regard as better suited for the current stage of development of the ecosystem of small to large enterprises which develop AIeMD, is through the means of relatively flexible guidelines to the MDR [4]. Either approach must be accompanied by focused targeted regulatory science on the best approach to the EU regulation of AIeMD. This is challenging to implement in a coordinated fashion in the European Union as there is no central regulatory body for medical devices with a research remit—the EMA has a highly limited role for medical devices, unlike for pharmaceuticals, where they have a broad remit to carry out regulatory research. A partial resolution of this issue could be achieved by means of a dedicated AIeMD expert panel. MDR requires the establishment of these EC-designated panels, with the remit of providing an opinion on the clinical evaluation of higher risk class medical devices in the European Union and of providing expert advice to the EC [14]. Currently, these panels are defined with medical disciplines, but it would be a timely and much-needed innovation to pilot the approach of an expert panel for the area of AIeMD and DHTs, to be the center of regulatory science expertise in this area for all stakeholders. The EU regulatory approach, with its distributed NB structure, is not well equipped to embark upon regulatory innovation, for example, through the useful mechanism of regulatory sandboxes. These are temporary approaches that facilitate the exploration of new regulatory approaches, generally for new technologies, with close interaction between developers and regulators. NBs do not have responsibilities in either regulatory policy development or the investigation of future policy through close interactions in real-world case studies, which is the function of sandboxes.

### US FDA Medical Device Regulation: Regulatory Science, Enforcement Discretion, and the AIeMD Action Plan

The Food and Drug Administration (FDA) approach to the introduction of AI-based DHTs regulation is markedly different to that of the European Union [4,45]. Comparison of the United States and European Union positions on the regulation of DHT and AIeMD needs to acknowledge the expressed will of recent US executives to promote digital health innovation, act in a manner that promotes growth, and take a relatively less stringent approach in its regulation, particularly of digital health innovations deemed to be of lower risk [11]. The FDA addresses the tensions and potential challenges by adopting a temporary hands-off enforcement discretion approach for large groups of use cases, when justified on an assessment of the risks. Practical guidelines and documents are also issued proactively and sufficiently early for regulatory readiness and are generally based on thorough research and stakeholder feedback [11]. There is an established process of sequential FDA regulation through a stepwise process of discussion documents and draft guidelines followed by finalized guidelines [11]. Change is not dependent on the buildup of NB capacity and NB adaptation to changes, as the system is not dependent on NBs, and there has been early and ongoing investment in regulatory IT systems, which are generally in place prior to the introduction of specific legislation that makes use of them [46]. In the United States, the latter are relatively better developed when compared to the European Union. In the opinion of the authors, these approaches leave the US legislators and the US FDA relatively less susceptible to regulatory failure through inadequate regulatory resourcing or connected to the readiness of IT systems.

### Enforcement Discretion

The FDA has adopted the highly flexible mechanism of enforcement discretion of some lower risk DHT and AIeMD. This approach has required no specific legislation, although its adoption has been accompanied by some controversy [11]. The effect of enforcement discretion on those products to which it applies is that the FDA does not intend to enforce requirements under the Federal Food, Drug, and Cosmetic Act (FD&C Act, as amended 1976) [47]. The FDA has since 2013 applied enforcement discretion to a large number of DHTs, which meet the definition of a medical device but which they consider to pose a lower risk to the public [48]. This is effectively a waiver of all FDA enforcement and registration requirements, with the justification that it frees up resources available for those DHTs that need more attention and that it allows the rapid technological and economic development of lower risk technologies. The degree to which the FDA is keeping the market under observation or how enforcement discretion may evolve was not described in detail in the guidance that introduced enforcement discretion. For this reason, it is advisable for manufacturers to develop all DHT close to the state of the art for medical devices, using a quality management system and closing any gaps to the standard of FDA approval as a medical device as quickly as possible, and to adopt good machine learning practice [4,45,49].

### Pre-Cert Pilot Program—A Regulatory Sandbox

In its Pre-Cert Program, the US FDA has sought to develop a new and pragmatic approach to DHT, which recognizes the unique characteristics of and marketplace for DHTs, so that they can continue to promote the innovation of high-quality, safe, and effective digital health devices [11]. This has the aim of developing, through industry partnerships, processes for facilitating DHT regulatory innovation, which can later be universally introduced and increase the speed of these technologies to the market. A DHT-specific approach to validation was developed, with a focus on the “culture of quality” of the applying companies. In September 2017, the FDA announced the 9 companies chosen to participate in the pilot program: Apple, Samsung, Verily, Pear Therapeutics, Tidepool, Phosphorus, Roche, Johnson & Johnson, and Fitbit [11]. Although this list includes a range of smaller companies and nonprofit organizations, overall, it is weighted toward large corporations. In our view, such programs should prioritize small- and medium-sized enterprises, which are the source of many of the innovations in AIeMD and DHT. The 2022 FDA report on the program concluded that the rapidly evolving technologies in the modern medical device landscape could benefit from a new regulatory paradigm and that this would require legislative change [50]. Although judged to have provided useful further data on this new US approach, the pilot faced challenges: (1) it was difficult to implement the pilot approaches without additional related statutory enforcement mechanisms or rights to information disclosure; (2) having only 9 participants led to few devices being available to the pilot; and (3) pilot device enrolment had to be further limited to prevent the knock-on effects of the program on potential third-party substantially equivalence devices.

### The Application of Regulatory Science—The Software as a Medical Device Action Plan and Research of Emerging Technologies

As a single federal regulatory body with a remit for leadership in the area of regulatory science, the FDA has been able to develop highly effective policies and structures in this area. There is a dedicated focus area for regulatory science, a plan for advancing regulatory science, and 4 associated academic Centers of Excellence in Regulatory Science and Innovation at leading universities in the United States [51]. One output of these structures is the FDA AI/machine learning–based software as a medical device (SaMD) action plan [4,45], which is an important example of the application of regulatory science principles: the FDA recognized AIeMD as an important emerging technology area that was not adequately dealt with under existing regulation, and this was followed by an intelligent and research-based development of purpose-designed regulatory approaches, with meaningful stakeholder engagement and dissemination.

The action plan recognizes that adaptive machine learning–based SaMD cannot be adequately regulated using pre-existing regulatory approaches. The action plan is an evolution of the FDA’s earlier premarket programs and the organization-based total product life cycle approach of the Pre-Cert Pilot Program. The approach is based on good machine learning practice principles [49]. Algorithm changes are transparently recorded for users, and robust approaches are included to minimize bias. A 2-component predetermined change control plan (PCCP) is envisioned, which includes (1) a SaMD prespecification that sets out the scope of permissible modifications and (2) an algorithm change protocol that sets out the methodology that will be used to implement the changes within the scope of the SaMD prespecification.

### How Should the United Kingdom Approach Post-Brexit Regulation

After implementing Brexit, the UK government has promised a proinnovation and flexible approach to regulating AI and envisages a light-touch, progrowth regulatory regime in its digital strategy [52-54]. The UK government plans to introduce a new post-Brexit UK Medical Device Regulation in 2023 and has completed a public consultation and published a response on this proposal [9]. At a high level, the United Kingdom has choices of whether to adopt very close convergence with the EU MDR, or with the FDA, or to explore the opportunities, and accept the complications and costs, of regulatory divergence. The US and EU experiences have fed into the approaches being developed in the United Kingdom but also constrain UK approaches as these are major export markets for UK manufacturers. It can choose between a relatively precautionary approach, as adopted by the European Union thus far and likely to continue under the EU AI Act [36], or exploring greater regulatory innovation and experimentation in its approach to DHT and AIeMD, which is similar to the US FDA [4,45].

### The UK Public Consultation and Response of the Government

The UK government/Medicines and Healthcare Products Regulatory Agency (MHRA) conducted a consultation with the public and with stakeholders between September and November 2021, a summary of the consultation was published on June 27, 2022, and a road map for SaMD and AIeMD was published on October 17, 2022 [9,55]. The summary document sets out the UK government’s response to specific aspects of the consultation, with 10% of content dedicated to AIeMD and DHT. Somewhat reassuringly, much of the UK government’s response is to set out pragmatic plans, which are aligned with the already-known positions of the International Medical Device Regulators Forum, the EU FDA, and the US FDA. This applies particularly to the areas of risk classification, premarket essential or general safety and performance requirements, and the approach toward standard definitions. The consultation included a proposal on an “airlock classification rule,” that is, a provision that would allow for a temporary classification to be applied to some DHT (including AIeMD), which have an unclear risk profile, and which then would be accompanied by monitoring and restricting the DHT as if they were a high-risk device. The concept is to promote early market access for novel and innovative DHT while providing simultaneous enhanced measures to ensure the safety of patients in the period until the risks of the device are fully understood. The UK government has put this concept on hold but intends to explore it further through subsequent consultations.

Generally, the UK government has opted for an approach that does not specify requirements in detail for AIeMD. Instead, the regulation will take a very similar approach to the EU MDR and will provide only high-level general requirements. The most innovative section of the guidance and UK governance position relates to the proposal to introduce PCCPs, concepts first introduced by the FDA [4,45]. The UK government’s response states that “the proper interpretation of [change management] requirements is difficult to find in guidance [...] In light of this, a clear legislative foothold *to manage change for software is required.* Predetermined change control plans are one method to streamline these processes.” [9]. Notably, this is not expressed solely in the context of AIeMD, but rather to medical device DHTs in general. In other words, PCCPs are proposed both for changes in AIeMD prediction models and for general “agile” changes in software carried out by software developers, including those changes that could currently be considered “[...] ‘significant’ or ‘substantial’ changes.” This is a revolutionary and highly interesting approach. Further specific details are provided on the implementation of PCCPs in the United Kingdom: (1) the overall approach has been stated to be in collaboration with international partners (likely the FDA, with whom the MHRA has already collaborated in this area [49], and who proposed the PCCP approach); (2) PCCPs will initially be on a voluntary basis; (3) PCCPs will run alongside a “robust post market surveillance and MHRA market surveillance system that produces a strong and clear safety signal, allowing for quicker and thorough capture of adverse incidents for SaMD” [9]; and (4) similar to the FDA approach [4,45], real-world evidence will be foundational to the UK PCCP approach. Another strength of the UK approach thus far has been to begin the integration of the 2 separate state oversight systems for AIeMD market access, that is, the regulatory layer and the health technology assessment layer. There is interaction and common principles between the proposal for the new UK MDR [9], and NICE (the UK health technology assessment body) recently proposed “Evidence standards framework (ESF) for digital health technologies,” including for measuring changes in the performance of DHTs over time [56,57].

### Conclusions

Substantial experience has been acquired in the United States, in applying agile, “better regulation” and science-based principles to the area of AIeMD and DHT regulation. A balance is needed between partially conflicting stakeholder interests—but stakeholder interests are not as 2-sided as sometimes presented. Patient and societal interests need a regulatory system, which ensures oversight but also allows commerce and innovation. Evidence-based and “better” regulation approaches aid in reaching this balance. Precautionary approaches may be justified in limited circumstances, where there are no alternatives, but their broad application is not in the interest of patients, health care systems, or national economies, or conducive to the ongoing public and political support of regulation. The AIeMD and DHT sector would better deliver health care system benefits and safety in partnership with agile, targeted innovative, and proportionally reactive oversight by regulatory bodies. Regulation must be developed in a manner that can be practicably implemented by both manufacturers and regulatory bodies as both have responsibilities and are critical to the delivery of health care. The UK approach is to select novel and well-designed features of international regulation. It also includes many flexible and positive approaches, for example, regulatory sandboxes involving smaller enterprises. It therefore also has the consequence of having a degree of divergence from all other international approaches. This will likely create an interesting dichotomy of regulatory forces on UK developers of AIeMD and DHT solutions. The United Kingdom may become a highly interesting location to initially develop solutions and to release them on the United Kingdom market. However, developers may face surprisingly large hurdles in transitioning UK approval (ie, UK Conformity Assessed marking) to approvals for other markets (eg, CE marking for the European Union).

## Abbreviations

AI: artificial intelligence
AIeMD: artificial intelligence–enabled medical device
DHT: digital health technology
EC: European Commission
FDA: Food and Drug Administration (US)
IVDR: in vitro diagnostic devices regulation
MDR: medical devices regulation
MHRA: Medicines and Healthcare Products Regulatory Agency (UK)
NB: notified body
PCCP: predetermined change control plan
SaMD: software as a medical device
UKRI: UK Research and Innovation
